# Animal Models of Depression and Drug Delivery with Food as an Effective Dosing Method: Evidences from Studies with Celecoxib and Dicholine Succinate

**DOI:** 10.1155/2015/596126

**Published:** 2015-05-03

**Authors:** João P. Costa-Nunes, Brandon H. Cline, Margarida Araújo-Correia, Andreia Valença, Natalyia Markova, Oleg Dolgov, Aslan Kubatiev, Naira Yeritsyan, Harry W. M. Steinbusch, Tatyana Strekalova

**Affiliations:** ^1^Department of Neuroscience, School for Mental Health and Neuroscience, Maastricht University, Universiteitssingel 40, 6200 MD Maastricht, Netherlands; ^2^CBA, Faculdade de Ciencias, Universidade de Lisboa, Campo Grande, 1749-016 Lisboa, Portugal; ^3^Instituto de Higiene e Medicina Tropical, Universidade Nova de Lisboa, Rua da Junqueira 100, 1349-008 Lisboa, Portugal; ^4^INSERM U1119, Université de Strasbourg, Bâtiment 3 de la Faculté de Médecine, 11 rue Humann, 67000 Strasbourg, France; ^5^Chronic Diseases Research Center, Faculdade de Ciências Médicas, Universidade Nova de Lisboa, Campo Mártires da Pátria 130, 1169-056 Lisboa, Portugal; ^6^Faculdade de Medicina Veterinária, Universidade Técnica de Lisboa, Avenida da Universidade Técnica, 1300-477 Lisboa, Portugal; ^7^Institute of General Pathology and Pathophysiology, Russian Academy of Medical Sciences, Baltiyskaya 8, Moscow 125315, Russia; ^8^Institute of Physiologically Active Compounds, Russian Academy of Sciences, Severnii proesd 1, Chernogolovka, Moscow Region 142432, Russia; ^9^Anokhin Institute of Normal Physiology, Russian Academy of Medical Sciences, Baltiyskaya 8, Moscow 125315, Russia; ^10^Division of Cardiology, University Hospital Magdeburg, Leipziger Strasse 44, 39120 Magdeburg, Germany

## Abstract

Multiple models of human neuropsychiatric pathologies have been generated during the last decades which frequently use chronic dosing. Unfortunately, some drug administration methods may result in undesirable effects creating analysis confounds hampering model validity and preclinical assay outcomes. Here, automated analysis of floating behaviour, a sign of a depressive-like state, revealed that mice, subjected to a three-week intraperitoneal injection regimen, had increased floating. In order to probe an alternative dosing design that would preclude this effect, we studied the efficacy of a low dose of the antidepressant imipramine (7 mg/kg/day) delivered via food pellets. Antidepressant action for this treatment was found while no other behavioural effects were observed. We further investigated the potential efficacy of chronic dosing via food pellets by testing the antidepressant activity of new drug candidates, celecoxib (30 mg/kg/day) and dicholine succinate (50 mg/kg/day), against standard antidepressants, imipramine (7 mg/kg/day) and citalopram (15 mg/kg/day), utilizing the forced swim and tail suspension tests. Antidepressant effects of these compounds were found in both assays. Thus, chronic dosing via food pellets is efficacious in small rodents, even with a low drug dose design, and can prevail against potential confounds in translational research within depression models applicable to adverse chronic invasive pharmacotherapies.

## 1. Introduction

The challenge to propose new powerful therapeutics for neuropsychiatric disorders, including antidepressants, has raised important questions regarding the efficiency of preclinical approaches currently being used [1–3]. Numerous limitations of the models of human neuropsychiatric pathologies have been intensively discussed during the last years [4–6]. Apart from a general problem of translational research, basic practical issues with animal models of neuropsychiatric conditions, however seemingly trivial, can essentially affect the validity of preclinical models, yet these can be addressed and resolved.

As with translational models in small rodents, these issues concern laboratory and procedural settings in animal studies. A number of experimental conditions have been shown to result in potential confounds for the practical application of animal models. The principals of these factors are commonly considered to include the circadian phase of manipulations [7, 8], cage enrichment [9–11], lighting conditions [12, 13], handling [14–16], vibration [17], the adverse taste of food or water [18, 19], and presence of and manipulations by an experimenter [20, 21]. They are sometimes believed to result in the remarkable variability in results that are extensively reported by the literature [18, 22–24]. The method and duration of dosing of experimental animals are one of the important sources of such confounds [25–27].

Various types of invasive treatments in rodents were shown to induce pain, inflammation, and distress, despite the proper use of standardized methods of application, especially when prolonged dosing is employed [28–30]. Obviously, this raises issues that concern not only the quality of studies, in which such dosing methods are used, but also animal welfare and ethical aspects. Nonetheless, in many cases, long and invasive drug administration to small rodents is problematic to avoid. This often applies, for instance, when non-water-soluble compounds have to be chronically administered, for example, during experimental conditions for which the induction of a desirable syndrome in an animal and/or the occurrence of the therapeutic drug's effect require a long time. The latter experimental situations are particularly typical for testing drugs in rodent models of depression where, for example, the induction of some key elements of depressive syndrome may take 2–12 weeks [24, 31, 32] and the occurrence of an antidepressant's effect, with most of the classical antidepressants, develops after 3-4 weeks of treatment [31, 33, 34].

In order to avoid the negative effects of chronic invasive dosing on overall animal welfare and experimental outcomes from standard models of depression, we evaluated the efficacy of drug delivery via food pellets in mice. First, we studied the effects of a three-week daily intraperitoneal vehicle injection in the mouse forced swim test, a common scheme of testing for the antidepressant-like effects of various treatments [32, 34, 35]. As this manipulation resulted in an increase of floating scores, a measure of “behavioural despair,” indicating a “prodepressant” effect of daily intraperitoneal injections for the experimental animals, we probed an alternative way of dosing using food pellets.

Though drug delivery with voluntarily consumed food is one of the common methods of dosing, its use in laboratory research is quite limited. Meanwhile, in many cases, this mode of pharmacological treatment is seen as advantageous because it enables the maintenance of a steady blood concentration for the drug, in contrast to bolus drug administration. However, it is sometimes viewed as insufficiently reliable due to its reliance on food intake and the variable bioavailability of some compounds depending on their delivery route [36, 37].

However, given that, in a reasonable proportion of the experimental situations, the consummatory behaviour of laboratory animals is not altered and the standard pharmaca, whose bioavailability and metabolism are well known not to be sensitive to the treatment method, are used, dosing with the voluntary intake of food pellets can probably be exploited much more frequently. Apart from the obvious benefits of animal wellbeing, the delivery of investigational drugs with food pellets can increase the validity of translational models as it simulates a human equivalent therapeutic dosing route.

In this study, we first used, via food pellets, a low dose of a classical antidepressant reference drug, imipramine (7 mg/kg/day), for which chronic administration via drinking water for 3 weeks was recently reported to evoke an antidepressant effect in a model of stress-induced anhedonia [38]. A low dose of antidepressant was selected because we sought to evaluate the usefulness of this dosing method at the lowest possible dosage limit which is used in other means of drug administration and because imipramine may exert side-effects when applied in higher concentrations [6]. The effects of imipramine delivered with self-made food pellets were tested in the forced swim test as well as, in order to exclude potential nonspecific effects of treatment, in a battery of behavioural tests including dark/light box, O-maze, novel cage, open field, and two-bottle sucrose test. Finally, to verify the applicability of this defined dosing method with food pellets, we tested the effects of new potential antidepressants: celecoxib, a non-water-soluble compound, at the dose of 30 mg/kg/day, which was selected based on previously published data [39, 40], and dicholine succinate whose dose was applied at 50 mg/kg/day based on previous results [42], in the forced swim and tail suspension tests. Imipramine, applied at 7 mg/kg/day [6, 36, 41, 42], and citalopram, 15 mg/kg/day [6, 33, 38], were used as pharmacological references.

## 2. Materials and Methods

### 2.1. Animals and Housing

Three-month-old C57BL/6N male mice were supplied by Instituto Gulbenkian de Ciência, Oeiras, Portugal, and housed individually in standard laboratory conditions under a reverse 12:12 h cycle (lights on at 21:00). Behavioural tests took place from the onset of the dark phase of the light cycle (9:00 h). The testing was carried out in a dark quiet room in morning hours. All procedures were in accordance with the European Union's Directive 2010/63/EU, Portuguese Law-Decrees DL129/92 (July 6th), DL197/96 (October 16th), and Ordinance Port. 131/97 (November 7th). This project was approved by the Ethical Committee of the New University of Lisbon.

### 2.2. Study Flow with Chronic Intraperitoneal Injections

**Figure 1:**
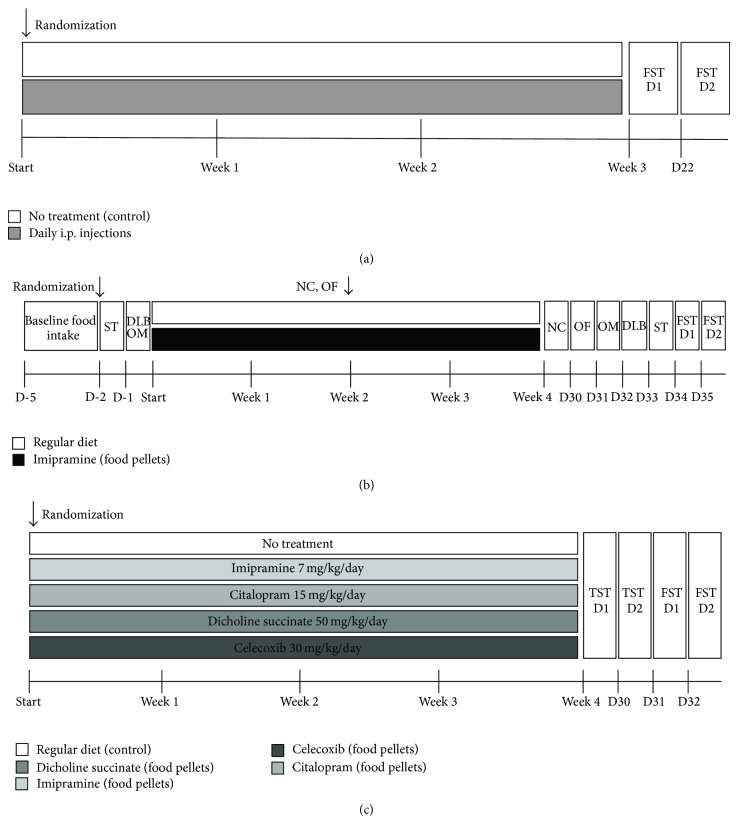
Experimental design. (a) Schematic timeline of studies with vehicle injections, chronic delivery with food pellets of (b) imipramine and (c) new drug candidates to antidepressants experiments. i.p.: intraperitoneal injection of a vehicle; FST: forced swim test; TST: tail suspension test; DLB: dark-light box; OM: O-maze; NCT: novel cage test; OF: open field; ST: sucrose test; d: days.

This study used a broadly applied treatment, in small rodents, of chronic intraperitoneal injections [30]. We have chosen to expose mice to a three-week daily intraperitoneal injections of NaCl at volume 0.01 mL/g body weight (for scheme of study flow, see Figure 1(a)). Control mice were not treated but handled daily. Starting from the next day after this period, mice were tested in the two-day forced swim test as previously described [38, 41]. Behavioural data were scored using Noldus EthoVision XT 8.5 (Noldus Information Technology, Wageningen, Netherlands). Number of mice per group is indicated in figure legend.

### 2.3. Study Flow with Chronic Imipramine Delivery via Food Pellets

As a next step, we exposed mice to self-made food pellets that contained imipramine for four weeks. Prior to starting treatment, animals were balanced upon body weight. The calculation of the used concentration of imipramine in food pellets was based on a daily food intake of experimental mice that constituted 2.89 ± 0.26 g and a desirable dosage of 7 mg/kg/day. The selection of this dose was based on previously obtained data that showed the efficacy of the dose [38] and a lack of such with chronic imipramine delivery via drinking water at a dose of 2.5 mg/kg in mice. Control mice received a regular diet. Before the start and after four weeks of dosing, all mice were tested in the sucrose preference test, O-maze test, and the dark/light box, as described elsewhere [43, 44]. After two and four weeks of dosing, locomotor activity of all mice was studied in the novel cage and open field tests, as described elsewhere [41, 42, 44]. At the end of behavioural testing, a two-day forced swim test with 6 min sessions was performed as previously described ([38, 41]; for scheme of study flow, see Figure 1(b)). Number of mice per group is indicated in figure legend.

### 2.4. Study Flow with Chronic Delivery via Food Pellets of New Candidates to Antidepressants

Next, we subjected mice to food pellets that contained imipramine, citalopram, celecoxib, or dicholine succinate for four weeks. Prior to starting treatment, animals were balanced upon body weight. The latter two drugs are regarded as compounds with potential antidepressant activity [39, 40, 42]. The calculation of drug concentrations was based on daily food intake of experimental mice, and desirable doses were 7 mg/kg/day, 15 mg/kg/day, 30 mg/kg, and 50 mg/kg, respectively. Control mice received regular diet. A two-day tail suspension test and a two-day forced swim test were carried out during four consecutive days after the termination of the dosing period, as described elsewhere [41, 43] (for scheme of study flow, see Figure 1(c)). Number of mice per group is indicated in figure legend.

### 2.5. Preparation of Pellets

Imipramine hydrochloride (Sigma-Aldrich, Munich, Germany), citalopram (Lundbeck, Copenhagen, Denmark), or celecoxib (Pfizer, Berlin, Germany) was added to commercial chow (Mucedola SRL, Milan, Italy) that was turned to powder by a blender. Small amounts of distilled water were added, and food pellets of a similar size to commercial pellets were formed and dried overnight (16 h) at 60°. New pellets were prepared twice a week in order to refresh the food supply of experimental groups. The content of drugs was adjusted to the dose indicated above and was based on the consumption of normal diet that was averaged over 3 days. Food pellets containing dicholine succinate (Buddha Biopharma Oy Ltd., Helsinki, Finland) were prepared in a similar way, using a 7% solution of the compound; the content of drug was adjusted to the abovementioned daily dose of this drug.

### 2.6. Behavioural Tests

#### 2.6.1. Forced Swim Test

The Porsolt forced swim test has been used as described elsewhere [38, 41]. Mice were subjected to two 6 min swimming sessions spaced 24 h apart in a transparent cylinder (*Ø* 17 cm) filled with water (+23°C, water height 13 cm, height of cylinder 20 cm, and illumination intensity 25 Lux). Floating behaviour was defined by the absence of any directed movements of the animals' head and body and was scored with Noldus EthoVision XT 8.5 (Noldus Information Technology, Wageningen, Netherlands). Using this method, the latency of the first episode of floating and the duration of floating behaviour were recorded during the 6 min swimming session on Day 1 and Day 2 of the test. Latency to begin floating was scored as time between introduction of the animal into the pool and the first moment of complete immobility of the entire body for a duration of >3 seconds. The total time spent floating, number of floating episodes, mean velocity, and distance moved were scored for the entire duration of the test using posttest video footage.

#### 2.6.2. Dark/Light Box

The dark/light box (Technosmart, Rome, Italy) consisted of two plexiglass compartments, one black/dark (15 cm × 20 cm × 25 cm) and one lit (30 cm × 20 cm × 25 cm), connected by a tunnel. Anxiety-like behaviour was assessed by earlier validated measures [41, 43]. Mice were placed into the dark compartment, from where they could visit the lit box, illuminated by light of 25 Lux intensity. The latency of the first exit to the light compartment, the total duration of time spent in the lit box, and the number of visits to this anxiety-related compartment were scored by visual observation over 5 min.

#### 2.6.3. Elevated O-Maze

The apparatus (Technosmart, Rome, Italy), which consisted of a circular path (runway width 5.5 cm, diameter 46 cm), was placed 50 cm above the floor. Two opposing arms were protected by walls (height 10 cm), and the illumination strength was 25 Lux. The apparatus was placed on a dark surface in order to reduce reflection and maintain control over lighting conditions during testing. Anxiety-like behaviour was assessed using previously validated parameters [38, 44]. Mice were placed in one of the closed-arm compartments of the apparatus. The latency of the first exit to the anxiety-related open compartments of the maze, the total duration of time spent therein, and the number of exits to the open arms were scored during a 5 min observation period.

#### 2.6.4. Novel Cage Test

The novel cage test was performed to assess vertical activity, as described elsewhere [38]. Mice were introduced into a standard plastic cage the size of their home cage filled with small amounts of fresh sawdust. The number of exploratory rearings was counted under red light during a 5 min period.

#### 2.6.5. Open Field

The open field apparatus consisted in four square arenas (50 cm × 50 cm × 50 cm), made of wood covered by white Resopal. Mice were put in the center and their behaviour was recorded on camera for 10 min. The open field was illuminated with white light (25 Lux). Distance moved and mean instant speed were analysed off-line using the Any-maze software (Stoelting Co., Wood Dale, IL, USA), as described elsewhere [45].

#### 2.6.6. Sucrose Test

Animals were given 8 hours of free choice between two bottles of either 1% sucrose or normal drinking water, as described elsewhere [38]. At the beginning and end of the period, the bottles were weighed and consumption was calculated. The beginning of the test started with the onset of the dark (active) phase of animals' cycle. To prevent the possible effects of side-preference in drinking behaviour, the position of the bottles in the cage was switched at 4 hours, halfway through testing. No previous food or water deprivation was applied before the test. The 1% sucrose solution is used in tests performed across the experiment. Percentage preference for sucrose is calculated using the following formula:

(1) Sucrose  Preference  =VSucrose  solutionVSucrose  solution+VWater×100%.

#### 2.6.7. Tail Suspension Test

The protocol used in this study was adapted from a previously proposed procedure [41, 43]. Mice were subjected to the tail suspension by being hung by their tails with adhesive tape to a rod 50 cm above the floor for 6 min. Animals were tested in a dark room where only the area of the modified tail suspension construction was illuminated by a spotlight from the ceiling; the lighting intensity on the height of the mouse position was 25 Lux. The trials were recorded by a video camera positioned directly in front of the mice while the experimenter observed the session from a distance in a dark area of the experimental room. This procedure was carried out twice with a 24 h interval between tests. The latency of the first episode of immobility, the total duration of this behaviour, and mean velocity were scored using Noldus EthoVision XT 8.5 (Noldus Information Technology, Wageningen, Netherlands) according to the protocol that was previously validated [41]. In accordance with the commonly accepted criteria of immobility, the immobility behaviour was defined as the absence of any movements of the animals' head and body. The latency of immobility was determined as the time between the onset of the test and the first bout of immobility.

### 2.7. Statistical Analysis

Data were analysed with GraphPad Prism version 5.00 for Windows (San Diego, CA, USA). Two-tailed unpaired *t*-tests were applied for two-group, two-tailed comparisons of independent data sets, as the distribution was normal. One-way ANOVA was used followed by a post hoc Dunnett for a comparison of more than two groups with a control; repeated measures ANOVA was used for analysis of repeated measures. The level of confidence was set at 95% (*P* < 0.05) and data are shown as mean ± SEM.

## 3. Results and Discussion

### 3.1. Effects of 3-Week Intraperitoneal Vehicle Injections on Floating Behaviour

**Figure 2:**
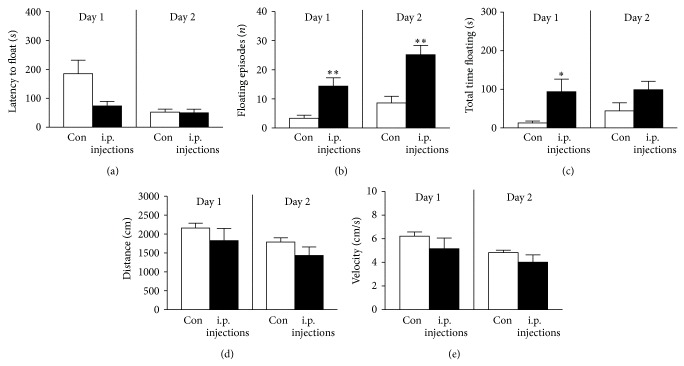
Chronic intraperitoneal injections increase depressive-like behaviour in the forced swim test. In comparison with control group, mice that received daily intraperitoneal saline injections over the course of three weeks had a nonsignificant reduction of the latency of floating (a) and significant increase of the total number of floating episodes (b) and total time spent floating (c). There were no significant differences in mean velocity (d) or distance swum (e) between control and injected groups. ^∗^ *P* < 0.05; ^∗∗^ *P* < 0.01 versus control (unpaired *t*-test). Con: control group (*n* = 7); i.p. injection: a group of mice subjected to intraperitoneal injections with a vehicle (*n* = 5). All data are means ± SEM.

Behaviour analysis revealed that animals subjected to injections displayed a nonsignificant decrease of latency to float as compared to control animals (Day 1: *P* = 0.11, *t* = 1.73; Day 2: *P* = 0.91, *t* = 0.12, Figure 2(a), unpaired two-tailed *t*-test). The number of floating episodes and the duration of floating in the chronically injected group were significantly higher than in control animals (Day 1: *P* = 0.0037, *t* = 3.89 and *P* = 0.016, *t* = 3.06; Day 2: *P* = 0.0016, *t* = 4.30 and *P* = 0.11, *t* = 1.77, resp.; Figures 2(b) and 2(c), unpaired two-tailed *t*-test); mean velocity of swimming and distance moved were nonsignificantly decreased (Day 1: *P* = 0.25, *t* = 1.25 and *P* = 0.31, *t* = 1.09; Day 2: *P* = 0.18, *t* = 1.43 and *P* = 0.15, *t* = 1.56, resp.; Figures 2(d) and 2(e), unpaired two-tailed *t*-test). This suggests increased “behavioural despair,” a sign of depressive-like state, in mice that received chronic manipulations with intraperitoneal injections.

Similar results were obtained in our previous experiments which demonstrated that three-and four-week daily injections in chronically stressed mice increased the number of individuals exhibiting signs of anhedonia, a reduced sensitivity to reward, in a sucrose preference test [18, 24]. Other studies showed that chronic intraperitoneal injections in rats evoke ultrasonic vocalizations at 22 kHz range, indicative of a negative emotional state that was reduced by preexposure of experimental animals to handling [16]. These “prodepressive” like changes found in this study could be potentially induced by well-recognized pathogenetic elements of depression, such as stress of manipulation [46] and pain experience [47, 48] and repeated situations of unescapable stress and helplessness [49], as well as inflammation [50].

### 3.2. Effects of Chronic Imipramine Delivery via Food Pellets on Floating Behaviours and Other Variables

**Figure 3:**
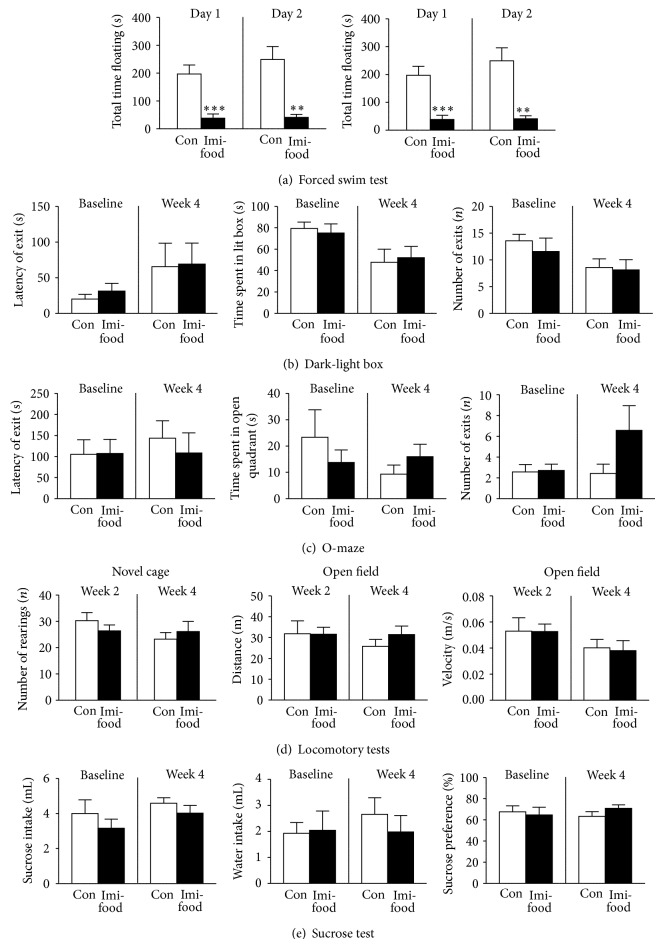
Effects of dosing with imipramine via food pellets on floating scores and other behaviours. (a) In comparison with control group, mice that received imipramine in food pellets over four weeks showed an increase of latency to float and total duration of floating in the forced swim test. No significant differences between the groups were found in parameters of anxiety (b) in the dark-light box and (c) O-maze tests. (d) Control and imipramine-treated mice showed similar numbers of rearings in the novel cage, distance travelled, and mean velocity in the open field test. (e) In the two-bottle sucrose preference test, water intake, sucrose solution intake, and sucrose preference were not different between imipramine-treated and control groups. ^∗∗^ *P* < 0.01, ^∗∗∗^ *P* < 0.001 versus control (unpaired *t*-test). Con: control group (*n* = 7); Imi-food: imipramine-treated group (*n* = 8). All data are means ± SEM.

In order to assess the efficacy of an alternative chronic dosing design that could preclude the adverse changes in behaviour described above, we evaluated the effects of four-week dosing of imipramine via food pellets in the forced swim test and supplementary behavioural paradigms. Animals subjected to imipramine treatment showed a significant increase in the latency to float and decreased immobility time, when compared to control animals (Day 1: *P* = 0.0002, *t* = 5.19 and *P* = 0.0008, *t* = 4.42; Day 2: *P* = 0.18, *t* = 1.43 and *P* = 0.0011, *t* = 4.28, resp.; Figure 3(a), unpaired two-tailed *t*-test). Thus, an applied low dose of antidepressant treatment delivered with food pellets induced an antidepressant-like effect in the present study.

This result is in line with our previous findings that showed that a 3-week low dose administration of imipramine to C57BL6J mice via drinking water reduced such depressive symptoms as stress-induced decease in sucrose intake and preference, hyperlocomotion, and elevated aggressive behaviour [38]. Similar behavioural results were obtained in the chronic stress depression model with CD1 mice [42] and in a model of elderly depression in 18-month-old C57Bl6N mice [41]. The low dose imipramine antidepressant effects were accompanied by preservation of normal activity of brain peroxidation enzymes which were suppressed by chronic stress [38]. These effects are typical for antidepressant effect manifestations induced by tricyclics in rodents [35, 51].

Further, in order to rule out potential effects of imipramine administration on anxiety, locomotion, and liquid intake that were previously reported in mice treated with this drug at a dose of 15/mg/kg in C57BL6N mice, we performed supplementary tests in all mice. In both anxiety paradigms, dark-light box and O-maze, animals treated with imipramine showed no significant differences in their behaviour from the control group: in latency of the exit to the anxiety-related areas, lit box and open arms (*P* = 0.94, *t* = 0.08 and *P* = 0.59, *t* = 0.55, resp.; unpaired two-tailed *t*-test), time spent in the lit box and open arms (*P* = 0.80, *t* = 0.26 and *P* = 0.28, *t* = 1.14, resp.; unpaired two-tailed *t*-test), and numbers of exits to these zones (*P* = 0.87, *t* = 0.17 and *P* = 0.13, *t* = 1.63, resp.; unpaired two-tailed *t*-test, Figures 3(b) and 3(c)). In locomotory tests, in comparison with control mice, animals treated with imipramine exhibited normal vertical activity, as shown by the number of rearings in novel cage (Week 2: *P* = 0.33, *t* = 1.01; Week 4: *P* = 0.54, *t* = 0.63, unpaired two-tailed *t*-test), as well as unchanged horizontal locomotion in the open field. In the latter test, no difference between groups was found in distance travelled (Week 2: *P* = 0.97, *t* = 0.038; Week 4: *P* = 0.31, *t* = 1.05) or mean instant velocity (Week 2: *P* = 0.98, *t* = 0.026; Week 4: *P* = 0.84, *t* = 0.21; Figure 3(d), unpaired two-tailed *t*-test). In a two-bottle sucrose preference test, there were no significant differences in water intake, sucrose solution intake, and sucrose preference between the groups (*P* = 0.47, *t* = 0.75; *P* = 0.32, *t* = 1.04; *P* = 0.20, *t* = 1.35, resp.; unpaired two-tailed *t*-test, Figure 3(e)). Finally, body weight was not different between control and imipramine-treated groups (*P* = 0.20, *t* = 1.37, data not shown, unpaired two-tailed *t*-test). There is no statistical significance using repeated measures ANOVA (data not shown).

Thus, the employed dosing with imipramine did not affect basic physiological variables, such as locomotion, liquid consumption, and body weight. Also, it did not affect the parameters of anxiety and sucrose ingestion, as reported in some studies that employ higher amounts of tricyclics [6, 31, 52, 53]. These results suggest that low dose imipramine treatment via voluntary food pellet intake can serve as an optimal pharmacological reference in animal models of depression that require prolonged antidepressant treatment of small rodents.

### 3.3. Effects of Chronic Delivery via Food Pellets of New Candidates to Antidepressants in the Forced Swim and Tail Suspension Tests

Next, we sought to investigate whether the defined method of antidepressant dosing with food pellets can be applicable with the testing of new drug candidates, one of which, celecoxib, is not soluble in water and, therefore, is problematic to deliver to the animals chronically. As such, we exposed a cohort of animals to food pellets containing new drug candidates: dicholine succinate or celecoxib. In addition, we used imipramine or citalopram as the antidepressant references.

**Figure 4:**
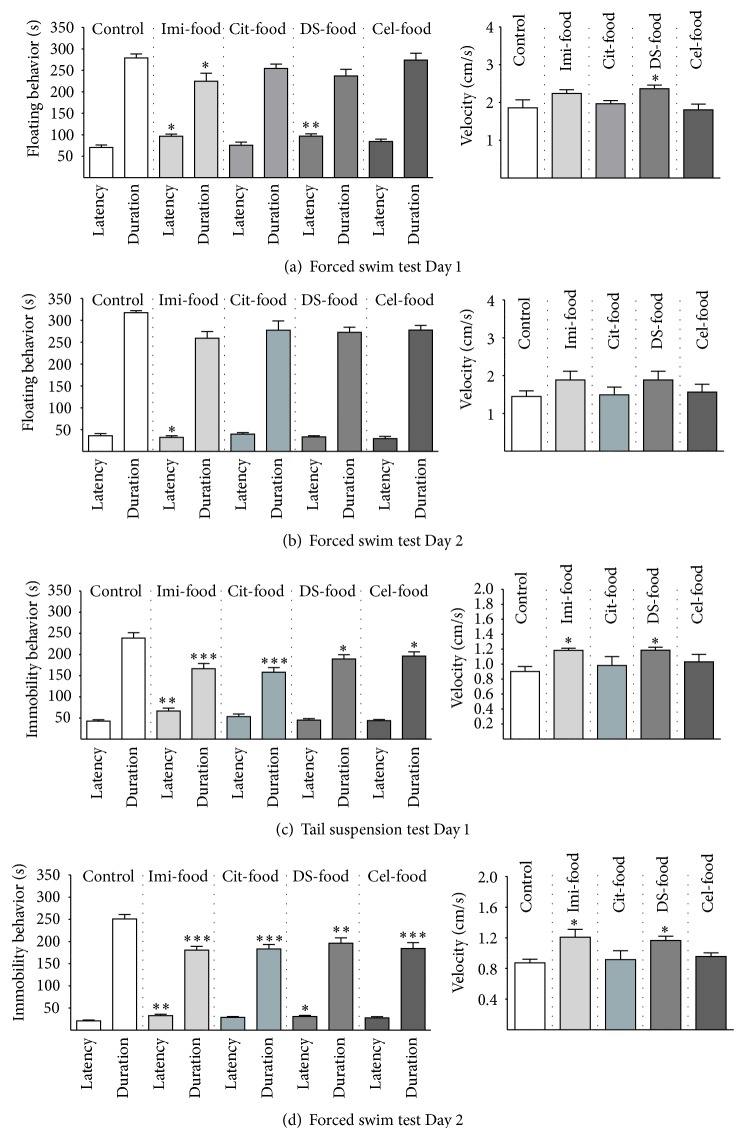
Effects of chronic delivery via food pellets of new candidates to antidepressants in the forced swim and tail suspension tests. (a) On Day 1 of the forced swim test, as compared with control, imipramine-treated animals elicited an increase in latency to float and reduced time spent floating, and dicholine succinate-treated groups displayed an increased swim velocity. (b) On Day 2 of the forced swim test, imipramine-treated group had higher latency to float in comparison to control mice; no other differences between treated and control groups were found. (c) On Day 1 of the tail suspension test, there was a significant increase of the latency of immobility and velocity in imipramine- and dicholine succinate-treated groups, as compared to controls. All treated groups showed a significant reduction of total time spent immobile, as compared to control animals. (d) On Day 2 of the tail suspension test, in comparison to control group, an increase of the latency of immobility was found in imipramine-treated group and an increase of velocity was observed in both imipramine- and dicholine succinate-treated mice. All animals that received a treatment demonstrated a significant reduction of total time spent immobile, in comparison to control group. ^∗^ *P* < 0.05, ^∗∗^ *P* < 0.01, and ^∗∗∗^ *P* < 0.001 versus control (one-way ANOVA with Dunnett* post hoc* tests). All groups were *n* = 10. Con: control group; Imi-food: imipramine-treated group; Cit-food: citalopram-treated group; DS-food: dicholine succinate-treated group; Cel-food: celecoxib-treated group. All data are means ± SEM.

In the forced swim test, one-way ANOVA revealed significant differences between the groups in the latency to float, total time spent floating, and velocity (Day 1: *P* = 0.0054, *F* = 4.24; *P* = 0.049, *F* = 2.60; and *P* = 0.22, *F* = 3.18, resp., Figure 4(a)). Post hoc Dunnett test showed that, on Day 1, in comparison with the control group, the latency to swim was increased in animals treated with imipramine or dicholine succinate (*P* < 0.05, *q* = 3.17 and *P* < 0.01, *q* = 3.20), the duration of immobility was decreased in the imipramine-treated animals (*P* < 0.05, *q* = 2.67), and velocity was elevated in the dicholine succinate-treated group (*P* < 0.05, *q* = 2.62). On Day 2 of the forced swim test, one-way ANOVA showed a trend to a statistically significant difference in the latency of floating and no differences in the duration of floating or velocity (*P* = 0.059, *F* = 2.46; *P* = 0.48, *F* = 0.89; and *P* = 0.40, *F* = 1.04, resp., Figure 4(b)). Dunnett post hoc test revealed a significant increase in latency to float in the imipramine-treated group (*P* < 0.05; *q* = 2.96). As a reduction of the parameters of floating behaviour in the forced swim test is a well-established measure of antidepressant activity of various compounds [32, 35], these data suggest that the applied treatment with imipramine or dicholine succinate induces an antidepressant effect and that the employed dosing was effective.

With the tail suspension test, one-way ANOVA showed that, on Day 1, there were statistically significant differences between the groups in the latency and duration of immobility (*P* = 0.007, *F* = 4.09; *P* < 0.0001, *F* = 3.43; and *P* < 0.0001, *F* = 3.43, resp., Figure 4(c)); a strong tendency to differences in velocity was found (*P* = 0.0505, *F* = 2.57). Post hoc Dunnett test revealed a significant difference in the latency of immobility from the control group in imipramine-treated animals (*P* < 0.01, *q* = 3.380) but not in other treatment groups. All groups that received pharmacological treatment had significantly reduced duration of immobility in comparison to control mice (imipramine-treated: *P* < 0.001, *q* = 4.50; citalopram-treated: *P* < 0.001, *q* = 5.02; dicholine succinate-treated: *P* < 0.05, *q* = 3.07; celecoxib-treated: *P* < 0.05, *q* = 2.65; Dunnett test). In comparison to control group, velocity was significantly increased in imipramine- and dicholine succinate-treated groups (*P* < 0.05, *q* = 2.55; *P* < 0.05, *q* = 2.56, resp.; Dunnett test).

On Day 2 of the tail suspension test, statistically significant differences between the groups were found in the latency of immobility, the duration of immobility, and velocity (*P* = 0.0159, *F* = 3.43; *P* < 0.0002, *F* = 6.90; and *P* = 0.012, *F* = 3.65, resp., one-way ANOVA; Figure 4(d)). Dunnett post hoc test showed a significant increase of the latency of immobility in imipramine- and dicholine succinate-treated animals, as compared with controls (*P* < 0.01, *q* = 3.48 and *P* < 0.05, *q* = 2.75, resp.). All treated groups had significantly reduced duration of immobility, as compared with control mice (imipramine-treated: *P* < 0.001, *q* = 4.46; citalopram-treated: *P* < 0.001, *q* = 4.31; dicholine succinate-treated: *P* < 0.01, *q* = 3.39; celecoxib-treated: *P* < 0.001, *q* = 4.24; Dunnett test). Velocity was significantly increased in comparison with control mice in the imipramine- and dicholine succinate-treated groups (*P* < 0.05, *q* = 2.97; *P* < 0.05, *q* = 2.59, resp.; Dunnett test). Since a decrease of immobility behaviour in the tail suspension test is generally considered as a manifestation of the antidepressant activity of various treatments [32, 54], these results evidence an antidepressant-like effect of the applied drugs and again the efficacy of the tested method of drug administration.

## 4. Conclusions

Thus, as a desirable alternative to invasive dosing, such as chronic intraperitoneal injections, the administration of various drugs via food pellets can be very efficient. The results from our study are in line with other successful attempts to avoid adverse drug delivery methodologies in translational research that showed, for example, the efficacy of treatment with analgesic therapy delivered via food in rats which were subjected to surgery [55]. The use of such methods could be particularly needed when repeated drug administration to stressed, operated, or immunodeficient laboratory animals is necessary and therefore could greatly improve not only animal welfare but also the validity of animal models.

## References

[1] Munos B. (2009). Lessons from 60 years of pharmaceutical innovation. *Nature Reviews Drug Discovery*.

[2] Pigott H. E., Leventhal A. M., Alter G. S., Boren J. J. (2010). Efficacy and effectiveness of antidepressants: current status of research. *Psychotherapy and Psychosomatics*.

[3] Araragi N., Lesch K.-P. (2013). Serotonin (5-HT) in the regulation of depression-related emotionality: insight from 5-HT transporter and tryptophan Hydroxylase-2 knockout mouse models. *Current Drug Targets*.

[4] Insel T. R., Sahakian B. J. (2012). Drug research: a plan for mental illness. *Nature*.

[5] Insel T. R. (2007). From animal models to model animals. *Biological Psychiatry*.

[6] Strekalova T., Anthony D. C., Dolgov O. (2013). The differential effects of chronic imipramine or citalopram administration on physiological and behavioral outcomes in naïve mice. *Behavioural Brain Research*.

[7] Sato Y., Seo N., Kobahashi E. (2005). The dosing-time dependent effects of intravenous hypnotics in mice. *Anesthesia and Analgesia*.

[8] Beeler J. A., Prendergast B., Zhuang X. (2006). Low amplitude entrainment of mice and the impact of circadian phase on behavior tests. *Physiology and Behavior*.

[9] Frick K. M., Stearns N. A., Pan J.-Y., Berger-Sweeney J. (2003). Effects of environmental enrichment on spatial memory and neurochemistry in middle-aged mice. *Learning and Memory*.

[10] Wolfer D. P., Litvin O., Morf S., Nitsch R. M., Lipp H.-P., Würbel H. (2004). Laboratory animal welfare: cage enrichment and mouse behaviour. *Nature*.

[11] Munn E., Bunning M., Prada S., Bohlen M., Crabbe J. C., Wahlsten D. (2011). Reversed light-dark cycle and cage enrichment effects on ethanol-induced deficits in motor coordination assessed in inbred mouse strains with a compact battery of refined tests. *Behavioural Brain Research*.

[12] Strekalova T., Spanagel R., Dolgov O., Bartsch D. (2005). Stress-induced hyperlocomotion as a confounding factor in anxiety and depression models in mice. *Behavioural Pharmacology*.

[13] Fukushiro D. F., Benetti L. F., Josino F. S. (2010). Environmental novelty and illumination modify ethanol-induced open-field behavioral effects in mice. *Pharmacology Biochemistry and Behavior*.

[14] Wahlsten D., Metten P., Crabbe J. C. (2003). A rating scale for wildness and ease of handling laboratory mice: results for 21 inbred strains tested in two laboratories. *Genes, Brain and Behavior*.

[15] Longordo F., Fan J., Steimer T., Kopp C., Lüthi A. (2011). Do mice habituate to “gentle handling”? A comparison of resting behavior, corticosterone levels and synaptic function in handled and undisturbed C57BL/6J mice. *Sleep*.

[16] Cloutier S., Wahl K., Baker C., Newberry R. C. (2014). The social buffering effect of playful handling on responses to repeated intraperitoneal injections in laboratory rats. *Journal of the American Association for Laboratory Animal Science*.

[17] Ringgold K. M., Barf R. P., George A., Sutton B. C., Opp M. R. (2013). Prolonged sleep fragmentation of mice exacerbates febrile responses to lipopolysaccharide. *Journal of Neuroscience Methods*.

[18] Strekalova T. V., Cespuglio R., Koval'zon V. M. (2008). Depressive-like state and sleep in laboratory mice. *Zhurnal Vyssheǐ Nervnoǐ Deiatelnosti Imeni I P Pavlova*.

[19] Strekalova T., Steinbusch H. W. M. (2010). Measuring behavior in mice with chronic stress depression paradigm. *Progress in Neuro-Psychopharmacology and Biological Psychiatry*.

[20] Sorge R. E., Martin L. J., Isbester K. A. (2014). Olfactory exposure to males, including men, causes stress and related analgesia in rodents. *Nature Methods*.

[21] Bohlen M., Hayes E. R., Bohlen B., Bailoo J. D., Crabbe J. C., Wahlsten D. (2014). Experimenter effects on behavioral test scores of eight inbred mouse strains under the influence of ethanol. *Behavioural Brain Research*.

[22] Crabbe J. C., Wahlsten D., Dudek B. C. (1999). Genetics of mouse behavior: interactions with laboratory environment. *Science*.

[23] Mandillo S., Tucci V., Hölter S. M. (2008). Reliability, robustness, and reproducibility in mouse behavioral phenotyping: a cross-laboratory study. *Physiological Genomics*.

[24] Strekalova T., Couch Y., Kholod N. (2011). Update in the methodology of the chronic stress paradigm: internal control matters. *Behavioral and Brain Functions*.

[25] Wahlsten D., Rustay N. R., Metten P., Crabbe J. C. (2003). In search of a better mouse test. *Trends in Neurosciences*.

[26] Sousa N., Almeida O. F. X., Wotjak C. T. (2006). A hitchhiker's guide to behavioral analysis in laboratory rodents. *Genes, Brain and Behavior*.

[27] Azar T., Sharp J., Lawson D. (2011). Heart rates of male and female Sprague-Dawley and spontaneously hypertensive rats housed singly or in groups. *Journal of the American Association for Laboratory Animal Science*.

[28] Cloutier S., Newberry R. C. (2008). Use of a conditioning technique to reduce stress associated with repeated intra-peritoneal injections in laboratory rats. *Applied Animal Behaviour Science*.

[29] Thiele T. E., Navarro M. (2014). “Drinking in the dark” (DID) procedures: a model of binge-like ethanol drinking in non-dependent mice. *Alcohol*.

[30] Gärtner K., Buttner D., Dohler K., Friedel R., Lindena J., Trautschold I. (1980). Stress response of rats to handling and experimental procedures. *Laboratory Animals*.

[31] Willner P. (2005). Chronic mild stress (CMS) revisited: Consistency and behavioural-neurobiological concordance in the effects of CMS. *Neuropsychobiology*.

[32] Harro J. (2013). Animal models of depression vulnerability. *Current Topics in Behavioral Neurosciences*.

[33] Strekalova T., Gorenkova N., Schunk E., Dolgov O., Bartsch D. (2006). Selective effects of citalopram in a mouse model of stress-induced anhedonia with a control for chronic stress. *Behavioural Pharmacology*.

[34] Overstreet D. H. (2012). Modeling depression in animal models. *Methods in Molecular Biology*.

[35] Cryan J. F., Valentino R. J., Lucki I. (2005). Assessing substrates underlying the behavioral effects of antidepressants using the modified rat forced swimming test. *Neuroscience & Biobehavioral Reviews*.

[36] Pottenger L. H., Domoradzki J. Y., Markham D. A., Hansen S. C., Cagen S. Z., Waechter J. M. (2000). The relative bioavailability and metabolism of bisphenol A in rats is dependent upon the route of administration. *Toxicological Sciences*.

[37] Volvert M.-L., Seyen S., Piette M. (2008). Benfotiamine, a synthetic S-acyl thiamine derivative, has different mechanisms of action and a different pharmacological profile than lipid-soluble thiamine disulfide derivatives. *BMC Pharmacology*.

[38] Cline B. H., Anthony D. C., Lysko A. (2014). Lasting downregulation of the lipid peroxidation enzymes in the prefrontal cortex of mice susceptible to stress-induced anhedonia. *Behavioural Brain Research*.

[39] Myint A. M., Steinbusch H. W. M., Goeghegan L., Luchtman D., Kim Y. K., Leonard B. E. (2007). Effect of the COX-2 inhibitor celecoxib on behavioural and immune changes in an olfactory bulbectomised rat model of depression. *NeuroImmunoModulation*.

[40] Maciel I. S., Silva R. B. M., Morrone F. B., Calixto J. B., Campos M. M. (2013). Synergistic effects of celecoxib and bupropion in a model of chronic inflammation-related depression in mice. *PLoS ONE*.

[41] Malatynska E., Steinbusch H. W. M., Redkozubova O. (2012). Anhedonic-like traits and lack of affective deficits in 18-month-old C57BL/6 mice: Implications for modeling elderly depression. *Experimental Gerontology*.

[42] Cline B. H., Steinbusch H. W. M., Malin D. (2012). The neuronal insulin sensitizer dicholine succinate reduces stress-induced depressive traits and memory deficit: possible role of insulin-like growth factor 2. *BMC Neuroscience*.

[43] Markova N., Chernopiatko A., Schroeter C. A. (2013). Hippocampal gene expression of deiodinases 2 and 3 and effects of 3,5-diiodo-L-thyronine T2 in mouse depression paradigms. *BioMed Research International*.

[44] Costa-Nunes J., Zubareva O., Araújo-Correia M. (2014). Altered emotionality, hippocampus-dependent performance and expression of NMDA receptor subunit mRNAs in chronically stressed mice. *Stress*.

[45] Pawluski J. L., Valença A., Santos A. I. M., Costa-Nunes J. P., Steinbusch H. W. M., Strekalova T. (2012). Pregnancy or stress decrease complexity of CA3 pyramidal neurons in the hippocampus of adult female rats. *Neuroscience*.

[46] Silverman M. N., Sternberg E. M. (2012). Glucocorticoid regulation of inflammation and its functional correlates: from HPA axis to glucocorticoid receptor dysfunction. *Annals of the New York Academy of Sciences*.

[47] Miller L. R., Cano A. (2009). Comorbid chronic pain and depression: who is at risk?. *Journal of Pain*.

[48] Goffer Y., Xu D., Eberle S. E. (2013). Calcium-permeable AMPA receptors in the nucleus accumbens regulate depression-like behaviors in the chronic neuropathic pain state. *The Journal of Neuroscience*.

[49] Pryce C. R., Azzinnari D., Spinelli S., Seifritz E., Tegethoff M., Meinlschmidt G. (2011). Helplessness: a systematic translational review of theory and evidence for its relevance to understanding and treating depression. *Pharmacology and Therapeutics*.

[50] Dantzer R., O'Connor J. C., Lawson M. A., Kelley K. W. (2011). Inflammation-associated depression: from serotonin to kynurenine. *Psychoneuroendocrinology*.

[51] von Frijtag J. C., van den Bos R., Spruijt B. M. (2002). Imipramine restores the long-term impairment of appetitive behavior in socially stressed rats. *Psychopharmacology*.

[52] File S. E., Tucker J. C. (1986). Behavioral consequences of antidepressant treatment in rodents. *Neuroscience and Biobehavioral Reviews*.

[53] Mogensen J., Pedersen T. K., Holm S. (1994). Effects of chronic imipramine on exploration, locomotion, and food/water intake in rats. *Pharmacology Biochemistry and Behavior*.

[54] Cryan J. F., Mombereau C., Vassout A. (2005). The tail suspension test as a model for assessing antidepressant activity: review of pharmacological and genetic studies in mice. *Neuroscience and Biobehavioral Reviews*.

[55] Jessen L., Christensen S., Bjerrum O. J. (2007). The antinociceptive efficacy of buprenorphine administered through the drinking water of rats. *Laboratory Animals*.

